# Nivolumab-DTPA-Based PD-1 Imaging Reveals Structural and Pathological Changes in Colorectal Carcinoma

**DOI:** 10.3389/fbioe.2022.839756

**Published:** 2022-02-14

**Authors:** Danni Li, Xiao Li, Jian Yang, Zhang Shi, Lu Zhang, Rou Li, Ye Peng, Jiajun Liu, Changjing Zuo

**Affiliations:** ^1^ Department of Nuclear Medicine, Shanghai Changhai Hospital, Shanghai, China; ^2^ Department of Radiology, Shanghai Changhai Hospital, Shanghai, China

**Keywords:** programmed cell death protein 1, nivolumab, SPECT, T1-weighted imaging, colorectal carcinoma, imaging probe precursor

## Abstract

Programmed cell death protein 1 (PD-1) expression is considered a prognostic marker of tumor response to the immuno-blocking therapy. In this study, nivolumab was conjugated with diethylenetriamine pentaacetate (DTPA) via condensation reaction between amidogen and *p*-SCN-Bn-DTPA, which provided labeling sites for ^99m^Tc^4+^ or Gd^3+^ ions. SPECT and magnetic resonance T1 weighted imaging (T_1_WI) analyses were performed on mouse models of colorectal carcinoma expressing humanized PD-1 antigen. Furthermore, PD-1 expression in intestinal tracks was assessed by immunohistochemistry, and then compared with the imageological findings. Nivolumab-DTPA was synthesized with varying molar ratios and was labeled with Gd or ^99m^Tc with a chemical purity of 96.28 ± 1.16% and good stability. In SPECT images, lesions with high ^99m^Tc-DTPA-nivolumab uptake and relatively clear background were shown at 6 h. Thereafter, the suspected intestinal thickening in Gd-free T_1_WI was observed at 2 h after the addition of Gd-DTPA-nivolumab. Notably, the results of both SPECT and T_1_WI analyses were consistent with the postmortem examination and immunohistochemistry results (for linear correlation with target to non-target ratios, *R* ^2^ = 0.8038, *p* < 0.05). In conclusion, nivolumab-DTPA could act as a probe precursor for identifying PD-1-positive lesions, not only through integrating the advantages of immunohistochemistry and molecular imaging but also by providing a noninvasive method for monitoring systemic changes.

## Introduction

Until recently, Food and Drug Administration (FDA)-approved therapeutic agents targeting the programmed cell death protein 1 (PD-1)/programmed cell death ligand 1 (PD-L1) axis have achieved impressive results in clinical immune checkpoint inhibitor (ICI) treatments, and serve as a crucial treatment for increasing cancer types, such as advanced melanoma (MM), non-small cell lung cancer (NSCLC), and colorectal carcinoma (CRC) (Constantinidou et al., 2019). Despite the encouraging advances, some adverse events have been shown to occur during ICI treatments as well. For instance, some patients respond well to the ICI treatment, while others do not respond at all. Clinical failure in many patients is not solely due to the inability to induce immune reactivation, but rather to an imbalance between T-cell reactivation and tumor burden (Huang et al., 2017). In addition to the expanding research on immunoblocking therapy (IBT), the search for prognostic biomarkers and relevant detection methods is urgently required to effectively categorize patients who are eligible for IBT or not.

Several studies have previously demonstrated that antigen expression is a prognostic marker for the therapeutic response of PD-1/PD-L1-based IBT (Gandini et al., 2016; Herbst et al., 2016). Therefore, immunohistochemistry (IHC) assay based on needle biopsy or surgical resection is currently used as the primary method of PD-1/PD-L1 evaluation. However, due to the diversity of tumor microenvironment and the dynamic changes in PD-1/PD-L1 expression resulting from concomitant treatments (Lim et al., 2016; Thomas et al., 2019), the spatial and temporal limitations of IHC analysis lead to uncertainty in decision-making for carrying out IBT. As an alternative, molecular imaging of PD-1/PD-L1 expression could timely assist in analyzing tumor lesions and metastasis as a whole, providing reproducible and non-invasive systemic monitoring of PD-1/PD-L1 expression.

Recently, nuclear medical imaging techniques, including positron emission tomography (PET), single photon emission computed tomography (SPECT), and multimodal imaging, have been considered for assessing PD-1/PD-L1 expression of diverse tumor types (Bensch et al., 2018; Gao et al., 2020; Lv et al., 2020). In this regard, imaging probes have been found to play a key role in molecular imaging, where the specific polypeptides or antibodies are often utilized as the target molecule of probe precursor (Wei et al., 2020). It is notable that findings of quantitative analysis of PD-1/PD-L1 imaging closely correlate with IHC analysis, which further provide a more comprehensive understanding of systemic immunity, for example, ^89^Zr-C4 PET in evaluating PD-L1 expression of NSCLC and prostate cancer (Truillet et al., 2018), ^111^In-PDL1.3.1 SPECT in evaluating PD-L1 expression of breast cancer (Heskamp et al., 2015), and ^89^Zr-nivolumab PET in evaluating PD-1 expression of advanced NSCLC (Niemeijer et al., 2018). Furthermore, the clinical significance of immune-related molecular imaging was demonstrated in establishing the treatment and predicting the downstream response as well. For example, Xing et al. conducted a phase I trial in patients with NSCLC, suggesting that anti-PD-L1-sdAb SPECT/CT using ^99m^Tc-NM-01 could be used to closely monitor changes in PD-L1 expression during PD-L1 immunotherapy (Xing et al., 2019). By imaging NSCLC, advanced bladder cancer, and triple-negative breast cancer with ^89^Zr-Atezolizumab PET, heterogeneity varying within and among lesions, patients, and tumor types was demonstrated, and thus it formed the basis for establishing the clinical treatment scheme, and as a result, the predictive value of molecular imaging was higher than that of IHC or RNA-sequencing-based predictive biomarkers (Bensch et al., 2018).

Considering the clinical needs for combined therapy, especially when PD-1/PD-L1 IBT is combined with surgery or stereotactic radiotherapy (SRT), the need of structural imaging was raised. Therefore, in such cases, functional magnetic resonance imaging (MRI) is considered to provide more accurate anatomical information. For T1 weighted imaging (T_1_WI), the accurate outlining of antigen-positive lesions can be achieved by analyzing before-and-after images upon Gadolinium (Gd)-based probe injection. Accordingly, the multiparametric imaging and its superior soft tissue contrast resolution, MRI is best suited for evaluation of the abdomen, and higher resolution with more details can be achieved considering the multiple parameters of MRI scans. Above all, functional imaging and anatomical imaging developed on the basis of specific antibodies have promising application prospects in PD-1 evaluation and potentially can provide further benefits for IBT.

**FIGURE 1:**
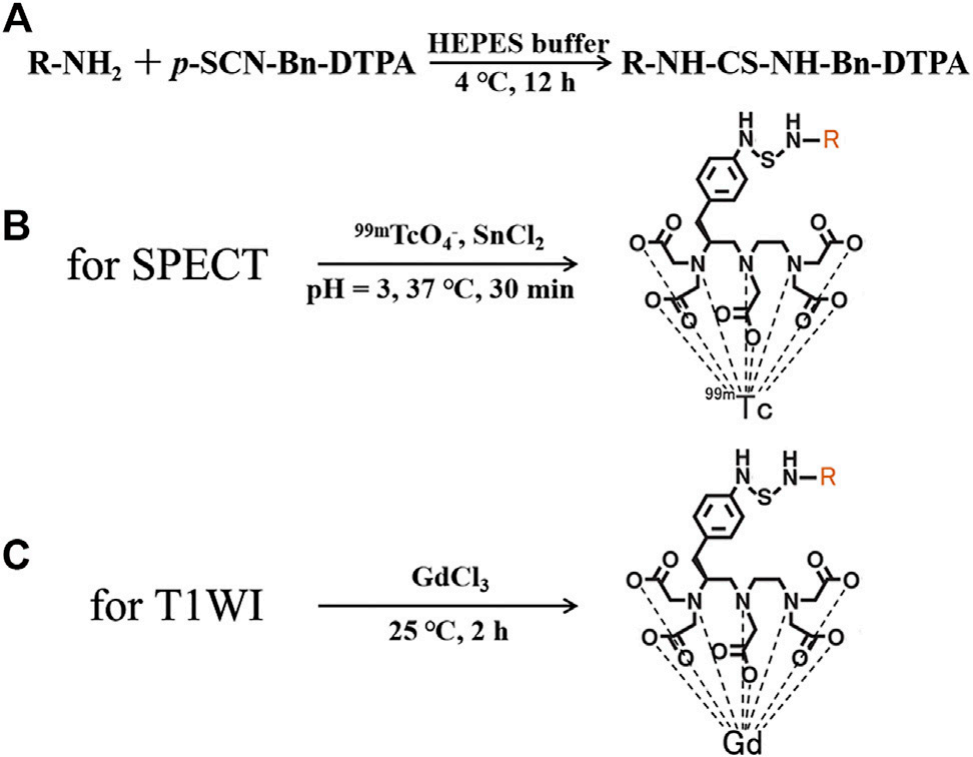
**(A)** Synthesis of nivolumab-DTPA, Structures of **(B)** ^99m^Tc-DTPA-nivolumab, and **(C)** Gd-DTPA-nivolumab.

As a clinically approved PD-1 antibody, nivolumab exhibits high affinity (K_d_ = 2.6 nM) and specificity for PD-1 antigen (Wang et al., 2014). In order to further expand the application of functional PD-1 imaging in the clinical research, nivolumab-DTPA was developed as an imaging probe precursor suitable for qualitative PD-1 assessment using Gd-based T_1_WI, and quantitative PD-1 assessment using Tc-99 m-based SPECT (Figure 1). Diagnostic values of the integrated functional imaging techniques were evaluated using the mouse models expressing humanized PD-1 antigen and bearing *in situ* colorectal carcinoma, which are necessary for clinical research and for a more comprehensive diagnosis based on combined therapy of PD-1 IBT and SRT.

## Materials and Methods

### Material and Reagents

Nivolumab was purchased from SelleckChem, and *p*-SCN-Bn-DTPA was purchased from Macrocyclics, Inc., Plano, Texas. ^18^F-FDG and ^99m^TcO_4_^-^ were purchased from Xinke Pharmaceutical Ltd., Shanghai, China. Gadolinium (III) chloride hexahydrate [GdCl_3_.6(H_2_O)] and stannous chloride [SnCl_2_.2(H_2_O)] were purchased from Adamas, Inc., CA, United States. Dialysis bags were purchased from Membrane Solutions, LLC, Shanghai, China. Other chemicals were purchased from Sigma-Aldrich China, Inc., Shanghai, China unless otherwise stated.

### Animals

Humanized PD-1 over-expressing (line name: C57BL/6J-Pdcd1^em1(hPDCD1)/Smoc^) female mice (6-weeks-old, 18–22 g) were purchased from Shanghai Model Organisms Center, Inc., Shanghai, China. Mice were raised under specific pathogen-free conditions. All the animal experiments were approved by the Ethics Committee of Shanghai Changhai Hospital and were conducted using the guidelines of ethical principles governing the animal welfare, rearing, and experimentation.

### Synthesis of Tracers

Nivolumab-DTPA was synthesized by conjugating nivolumab with the bifunctional chelator (BFC) *p*-SCN-Bn-DTPA. As shown in Figure 1, the isothiocyano group of *p*-SCN-Bn-DTPA was reacted with the amino group of nivolumab to form nivolumab-DTPA. In detail, *p*-SCN-Bn-DTPA was dissolved in DMSO at a concentration of 10 mg/ml. Around 500 µg of nivolumab (145 kDa) was dissolved in HEPES buffer and the pH was adjusted to 8. Thereafter, BFC was added in the solution at 4, 20, or 100 times more than nivolumab. Reaction was carried out at 4°C for 12 h. The excess *p*-SCN-Bn-DTPA was removed by using a 50 kDa millipore ultrafiltration tube.

For MRI probe preparation, 500 µg nivolumab-DTPA (nivolumab content) in PBS was reacted with GdCl_3_ at 100 M times more than nivolumab at 25°C for 2 h. Free Gd^3+^ ions were removed using a dialysis bag. In detail, a dialysis bag with a molecular weight cut-off of 20 kDa was used to seal the reaction system, and then placed in a beaker with distilled water. Magnetic stirring was used to promote ion exchange. Distilled water was changed every 4 h, and this operation was repeated 3 times to obtain the purified Gd-DTPA-nivolumab. For the measurement of nivolumab-DTPA with different DTPA ratios, 200 µg Gd-DTPA-nivolumab (nivolumab content) was ionized and dissolved in 100 μl, and then diluted to 1/10^4^ with distilled water. Around 100 µl solution was used to test the concentration of Gd^3+^ using inductively coupled plasma-mass spectrometry (ICP-MS; Perkin Elmer NexION 300D).

For SPECT probe preparation, newly prepared 111 MBq ^99m^TcO_4_^-^ was reduced by 20 µl SnCl_2_ (1 mg/ml, in HCl with pH = 3), and then was used to label 100 µg of nivolumab-DTPA at 37°C for 30 min. Radiochemical purity (RCP) was determined using a silica gel plate (solid phase) and acetone (mobile phase) system. Purified ^99m^Tc-DTPA-nivolumab was dissolved in 0.01 M PBS or 1% fetal bovine serum for the *in vitro* stability tests. The labeling rate and stability after 6 h at 37°C were measured by thin-layer chromatography (TLC) with a radioactive detector.

### *In vitro* Assay

Splenocytes were isolated from the spleens obtained from humanized PD-1 over-expressing mice following standard protocol. Referring to our previous labeling protocol (Li et al., 2018), ^125^I labeled Nivolumab and Nivolumab-DTPA, respectively. To perform co-culture, ^125^I-Nivolumab and ^125^I-Nivolumab-DTPA (0.37 MBq/µg) were added to the freshly isolated splenocytes (1×10^6^ cells) grown in 6-well plates, respectively. After incubation for 12 h at 4°C, cells were collected after rinsing three times with PBS. ^125^I radioactivity was determined in a gamma counter to evaluate their binding efficiency to PD-1.

### Single Photon Emission Computed Tomography/CT and MR Imaging

Four *in situ* CRC mouse models (highly expressed human PD-1 antigen) prepared using azoxymethane (AOM)/dextran sulfate sodium (DSS) chemical induction were used in this study. According to a previous study (Tanaka et al., 2003), AOM (10 mg/kg) was injected intraperitoneally. Seven days later, three cycles of feeding with water (2% DSS included) for 7 days, followed by pure water feeding for 17 days was followed. Thereafter, normal diet was followed, which lasted for another 50 days. Four mice without significant weight difference were randomly selected, and fluorine-18 fluorodeoxyglucose (^18^F-FDG) micro-PET/CT was performed to verify the successful preparation of the *in situ* CRC model. One of the above mice was sacrificed to evaluate the PD-1 expression of the observed foci by IHC analysis, and the other three were used for the subsequent imaging assays.

The imaging evaluation was as follows: 1) ^99m^Tc-DTPA-nivolumab (11.1 MBq/10 μg) was injected through the tail vein, and SPECT scan was performed 2 and 6 h after the injection. After an interval of 48 h, 2) T_1_WI was observed 2 h after the injection of 100 μg Gd-DTPA-nivolumab through the tail vein and was compared with the intestinal morphology and signals of the pre-injection image.

For SPECT/CT imaging, 50 μl lidocaine (3 wt%) was intraperitoneally injected before each scan. SPECT/CT (Symbia T16, Siemens, Erlangen, Germany) scans were performed to evaluate the distribution and metabolism of tracer. Scan parameters used were as follows: low-energy university collimator, matrix, 128 × 128; zoom, 2.67; energy peak, 140 keV; window width, 20%; and frames, 60 s/frame. CT scan: tube voltage, 130 kV; tube current, 35 mA; and slice thickness, 1 mm. For ^99m^Tc-nivolumab SPECT/CT images, suspicious tumor lesions with high tracer uptake were outlined, and radioactivity of both lesions and whole body were recorded.

For MRI scans, a Siemens scanner (Magnetom Trio 3.0T, Siemens Medical Solutions, Erlangen, Germany) with a hand coil was used. The scan parameters were as follows: for T_1_WI, TE, 13.8 ms; TR, 4,000 ms; slice space, 1 mm; slice thickness, 2 mm; FOV, 120 mm × 120 mm; matrix, 128 × 128; number of excitations, 1. For image reading of Gd-nivolumab T_1_WI, the anatomy structure of intestinal tracts was observed, and the images were compared with that of Gd-free T_1_WI to determine the signal enhancement resulting from tracer uptake.

After the acquisition of MRI, the mice were sacrificed to record the focal anatomy and evaluate PD-1 expression using IHC analysis. ImageJ software (NIH) was used to quantify positively stained areas of immunostaining images. SPECT and T_1_WI results were compared with that of IHC and visual observation to evaluate the diagnostic performance.

### Quantification and Statistical Analysis

Data, including the RCPs and biodistribution, were presented as the mean ± SD of at least N = 3. The related-coefficient test was used for data comparisons. Differences with *p*-values less than 0.05 were considered as statistically significant. Data were analyzed using SPSS v.23.0 for Windows (SPSS Inc., Chicago, IL, United States).

## Results

### Conjugation Efficiency of Nivolumab and *p*-SCN-Bn-DTPA

**FIGURE 2:**
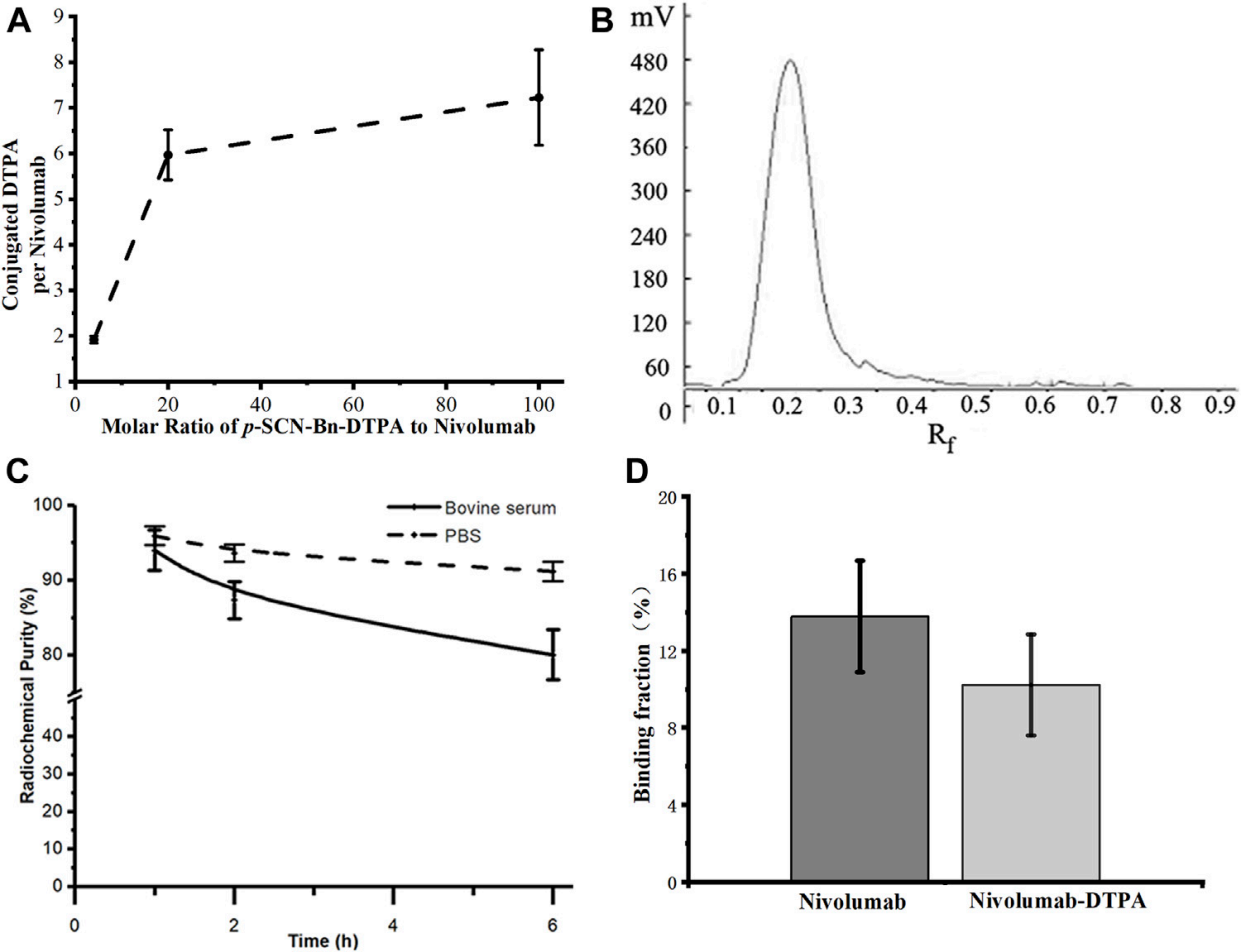
**(A)** Quantification of conjugated DTPA to nivolumab, **(B)** radioactive TLC spectrum of purified ^99m^Tc-labeled nivolumab, **(C)** the *in vitro* stability of ^99m^Tc-DTPA-nivolumab in 0.01 M PBS and fetal bovine serum concentration of 1% (w/w). **(D)** Validation of *in vitro* binding efficiency of Nivolumab and Nivolumab-DTPA to PD-1.

Due to the 1:1 correspondence between Gd and DTPA in Gd-DTPA-nivolumab, the molar ratio of DTPA to nivolumab was indirectly quantified through the quantification of labeled Gd on nivolumab-DTPA. For this, Gd-DTPA-nivolumab was dissociated and the free Gd was quantified using ICP-MS. The ratios of conjugated DTPA to nivolumab were 1.92 ± 0.08, 5.97 ± 0.55, and 7.23 ± 1.05, when the molar ratios of *p*-SCN-Bn-DTPA to nivolumab were 4, 20, and 100, respectively (Figure 2A). Considering that the conjugated DTPA reached the upper limit when the ratio of *p*-SCN-Bn-DTPA to nivolumab was 100, the ratio of 20 was used in the following steps as 5.97 ± 0.55 DTPA conjugated to per nivolumab. Additionally, a free Gd concentration below the ppm level provided *in vitro* stability to Gd-DTPA-nivolumab after 6 h at 37°C in 0.01 M PBS or in 1% fetal bovine serum.

### Radiochemical Purity and Stability of ^99m^Tc-DTPA-Nivolumab and Specific Binding Characteristics

The radiochemical purity of ^99m^Tc-DTPA-nivolumab was estimated as 96.28 ± 1.16% after purification (Figure 2B). *In vitro* stability studies at 37°C showed that 91.15 ± 1.26% stability was maintained after 6 h of incubation in 0.01 M PBS and 80.05 ± 3.35% stability was maintained after 6 h of incubation in 1% fetal bovine serum (Figure 2C). As shown in Figure 2D, the cell binding ratio of ^125^I-Nivolumab and ^125^I-Nivolumab-DTPA to PD-1 was 13.78 ± 2.90% and 10.22 ± 2.62%, respectively (*p* > 0.05). The similar PD-1-binding efficiency of Nivolumab and Nivolumab-DTPA indicated that conjugating of DTPA does not affect targeting of Nivolumab to PD-1 *in vitro*.

### Validation of the Tumor Model Mice With PD-1-Positive CRC

**FIGURE 3:**
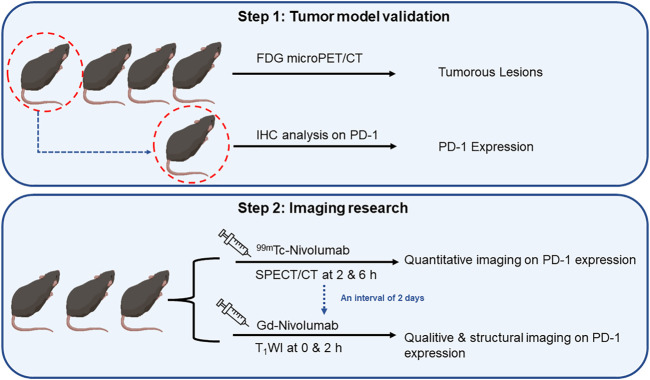
The flowchart of research on quantitative and qualitative PD-1 imaging, including the validation of tumor model mice and sequence of SPECT/CT and T_1_WI.

**FIGURE 4:**
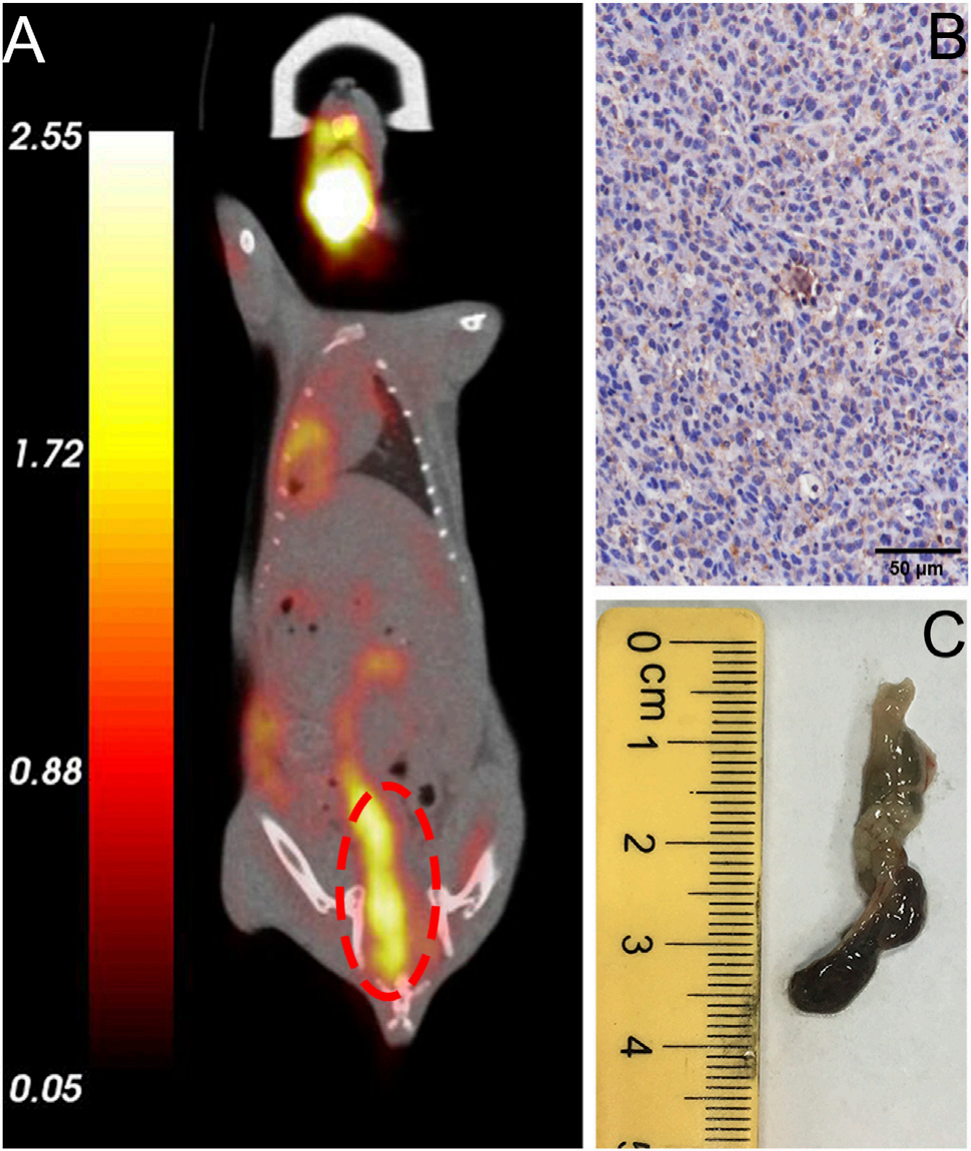
Validation of typical *in situ* colon tumor mice. **(A)** FDG micro-PET/CT image of a tumor mouse model showing a lesion at the end of the colon (marked in red circle), **(B)** IHC analysis revealed high expression of humanized PD-1, **(C)** Specimen of colorectal carcinoma that exhibited high FDG uptake.

All mice were validated using FDG microPET/CT scan, and one of them were sacrificed for evaluation of PD-1 expression by IHC analysis (step1 in Figure 3). For lesions detected on FDG PET/CT scan, hyperemia, edema, and nodules with high PD-1 expression were found in the corresponding intestinal tracts. The locally high uptake of ^18^F-FDG and PD-1-expressed tumor tissues indicated the successful establishment of animal models. Before subjecting to further imaging analysis (step2 in Figure 3), all mice were tested through FDG PET/CT scan, and the ones who fulfilled the criteria of locally high uptake of FDG were chosen. Approximately 70% (7/10) of mice developed with one or more primary lesions of colorectal carcinomas, and the distribution of lesions was random. A typical FDG PET/CT scan image of the mouse model and corresponding PD-1 IHC and postmortem examinations are shown in Figure 4.

### Qualitative PD-1 Imaging of the Tumor Model Mice

**FIGURE 5:**
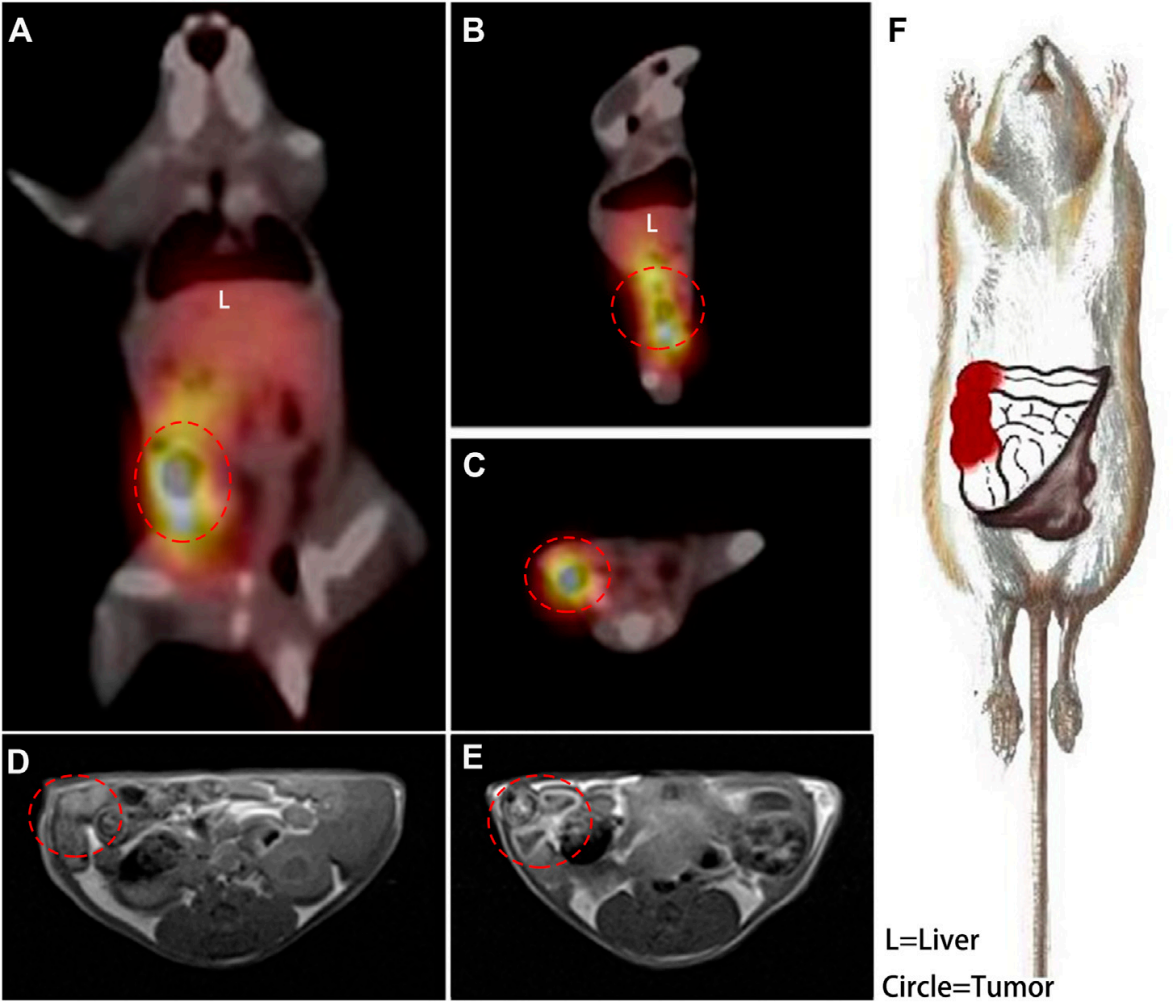
A coronal plane **(A)**, sagittal plane **(B)** and cross-section **(C)** SPECT images acquired at 6 h post injection of ^99m^Tc-DTPA-nivolumab, and the right colon with high tracer uptake is circled. In transverse T_1_WI **(D)**, the suspected thickened intestine is circled, and the enhanced intestinal wall is circled in an enhanced scan acquired at 2 h post injection of Gd-DTPA-nivolumab **(E)**. A detailed description of lesions with high PD-1 (in red) was drawn based on these functional imaging findings **(F)**.

Nivolumab-DTPA shared the same *in vivo* distribution. A series of typical imageological findings were presented in Figure 5. In SPECT/CT images, a focus with high ^99m^Tc-DTPA-nivolumab uptake was shown in the right colon at 6 h post injection, while a relatively clear background appeared without indicating any unusual high uptake. Conversely, SPECT images at 2 h post injection presented a high background in untargeted organs, such as the liver, heart, and abdomen, which shielding the colorectal lesions. In MR T_1_W images, suspected thickening of the intestinal wall in the right intestine was displayed. Furthermore, an enhanced scan image was acquired at 2 h after the injection of Gd-DTPA-nivolumab, which revealed a marked signal enhancement of the suspected thickening of the intestinal wall. Based on these imageological findings, a clear description of intestinal lesions with high PD-1 expression was obtained.

The findings based on Gd-DTPA-nivolumab T_1_WI and ^99m^Tc-DTPA-nivolumab SPECT/CT were found to be consistent with the postmortem examinations, in which congestion and necrosis of the intestinal lesion were detected. Hematoxylin-eosin (HE) staining of the intestinal tumor tissues in mice demonstrated that the heteromorphism of the cancer cells was evident, and the nucleoli were clearly observed. Further, it also indicated that the cancer cells grew densely. Quantitative analysis of the images and IHC are summarized next.

### Quantitative PD-1 Imaging of the Tumor Model Mice

**FIGURE 6:**
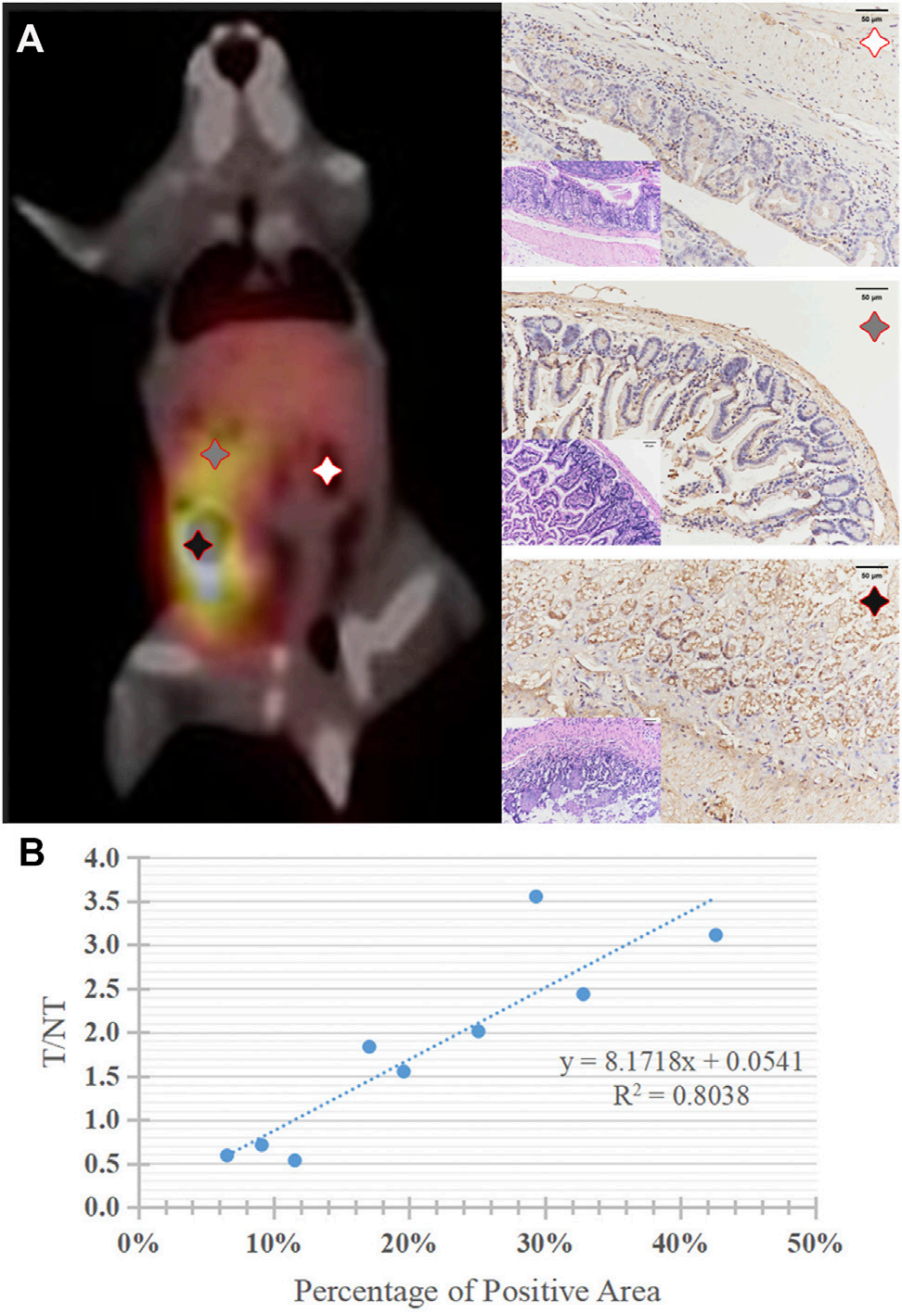
**(A)** A typical ^99m^Tc-DTPA-nivolumab SPECT/CT image of tumor mouse model, and corresponding histopathology (HE staining, × 200) and PD-1positive expression of lesions with high, medium, and very low ^99m^Tc-DTPA-nivolumab uptake, brownish yellow staining was observed on the surface of tumor cells (IHC, × 200). **(B)** The correlation of quantitative imaging (reflected by T/NT values) and IHC (reflected by the percentage of PD-1 positive areas).

Quantitative PD-1 imaging mainly relied on the ^99m^Tc-DTPA-nivolumab SPECT/CT, where the tumor to liver ratios of tracer uptake varied from 1.55 to 3.11. IHC results indicated that the intestinal tumor exhibited high PD-1 expression, with 17.05–42.62% positively stained areas, and that the PD-1 area of IHC staining linearly correlated with target to non-target (T/NT) ratios with *R* ^2^ = 0.8038 (*p* < 0.05). This linear dependence exists in both lesions and normal tissues with T/NT values less than 1 (Figure 6).

## Discussion

In our previous work, we evaluated the pharmacokinetic properties of I-125 labeled nivolumab using the nude mouse xenograft model of colorectal carcinoma. Since only the B and NK cells were used, we could prove PD-1 targeting partially (Li et al., 2018). In this study, the tumor growth and angiogenesis of *in situ* tumor models were almost achieved in the humanized mouse cancer model, and therefore, the results obtained for systemic uptake of antibodies-based tracers were more convincing.

Being different from the nuclear medicine imaging, the probe precursor of nivolumab-DTPA could be successfully and stably labeled with Tc-99m and Gd, thereby proving to be suitable for functional SPECT and structural T_1_WI MRI analyses to further evaluate PD-1 expression and to outline the fine anatomies with high PD-1 expression. Utilizing the additive information of T_1_WI, tumorous lesions could easily be distinguished from others with high PD-1 expression, facilitating the establishment of therapies, such as the combined therapy involving stereotactic radiotherapy and PD-1 blockade therapy. Nivolumab-DTPA was developed on the basis of a commercial IgG antibody with a stable structure and bio-specificity to PD-1. This method has been efficiently utilized in the development of PD-L1 imaging probe ^89^Zr-atezolizumab, which has shown significant clinical value (Bensch et al., 2018).

As Figure 5 shown, a hypointensity region was depicted on the right colorectal area on T_1_WI (Figure 5D), and an obvious hyperintensity signal region was shown in the same colorectal area on contrast T1W image (Figure 5E). It indicted that the enhancement region could be the high PD-1 expression in early imaging at 2 h. Meanwhile, although 6 h assessment was needed for a clear background, SPECT was used for the quantification and whole-body assessment. When compared with FDG PET/CT, ^99m^Tc-DTPA-nivolumab SPECT/CT integrated with Gd-DTPA-nivolumab T_1_WI focused on pathological changes, potentially decreasing the false-positive diagnosis resulting from inflammation. In clinical practice, imaging modalities can be selected on the basis of clinical needs. The limitation of this study is that the T_1_W sequence that has been used does not appear to be fat suppressed, which makes it difficult to assess the enhancement effect. The use of a 3D T_1_W sequence may be preferable in future studies.

## Conclusion

In this study, it was demonstrated that nivolumab-DTPA, as an imaging probe platform, could be used in qualitative imaging by Gd-based T_1_WI, and quantitative imaging by Tc-99 m-based SPECT. Also, we believe that integrated functional imaging may be helpful in accurately evaluating the systemic expression of PD-1 in colorectal carcinoma and other cancer types. Moreover, translational values of the antibody-coupled imaging precursor were indicated in diagnosing microscopic cancers and tracking metastasis through PD-1 expression in this study.

## Data Availability

The original contributions presented in the study are included in the article/supplementary files, further inquiries can be directed to the corresponding author.
